# Economic Evaluation Study (Cheer Compliant) Laser Prostatectomy for Benign Prostatic Hyperplasia: Outcomes and Cost-effectiveness

**DOI:** 10.1097/MD.0000000000002644

**Published:** 2016-02-08

**Authors:** Yu-Chao Hsu, Yu-Hsiang Lin, Chih-Yuan Chou, Chen-Pang Hou, Chien-Lun Chen, Phei-Lang Chang, Ke-Hung Tsui

**Affiliations:** From the Department of Urology and Medicine, Prostate Health Laser Center, Chang Gung Memorial Hospital, Chang Gung University, Taoyuan (Y-CH, Y-HL, C-PH, C-YC, C-LC, P-LC, K-HT) and Department of Urology, Show Chwan Memorial Hospital, Changhua, Taiwan (C-YC).

## Abstract

To determine which surgical treatment for lower urinary tract symptoms, which is suggestive of benign prostatic hyperplasia (BPH), is more cost-effective and yields a better patient's preference. Treatment outcome, cost, and perioperative complications to assess the treatment effectiveness of using laser prostatectomy as a treatment for BPH were investigated in this study.

This retrospective study included 100 patients who underwent transurethral resection of prostate (TUR-P) and another 100 patients who received high-powered 120 W (GreenLight HPS) laser prostatectomy between 2005 and 2011.

International Prostate Symptom Score and uroflow parameters were collected before the surgery and the uroflow and postvoiding residual volumes were evaluated before treatment and at 3, 6, 12, and 24 months after treatment. The results of 100 treatments after HPS laser prostatectomy were compared with the results of 100 patients who received TUR-P from the same surgeon. Complication rates and admission costs were analyzed.

From 2005 to 2011, 200 consecutive patients underwent endoscopic surgery. Study participants were men with BPH with mean age of 71.3 years old. The peak flow rate went from 8.47 to 15.83 mL/s for 3 months after laser prostatectomy. Laser therapy groups showed better improvement in symptom score, shortened length of stay, and quality of life score when compared with those of TUR-P procedures. The estimated cost for laser prostatectomy was high when compared with cost of any other TUR-P procedural option at Chang Gung Hospital (*P* = 0.001). All admission charges were similar except for the cost of the laser equipment and accessories (mainly the laser fiber) (*P* = 0.001). Due to this cost of equipment, it increased the total admission charges for the laser group and therefore made the cost for the laser group higher than that of the TUR-P group.

Perioperative complications, such as the need for checking for bleeding, urinary retention rate or urosepsis rate within 30 days after the surgery, held no significant differences between both groups.

Compared with alternative treatment options, laser prostatectomy of the prostate is clinically effective but yields a high cost of treatment for symptomatic BPH.

## INTRODUCTION

Evaluation of treatments for chronic health conditions, relative to the best way to use an available budget, requires studies of both effectiveness and cost-effectiveness over the long term. With an estimated prevalence of up to over 80% in men older than age 80, benign prostatic hyperplasia (BPH) is a common problem among elderly men.^1^ Benign prostatic hyperplasia can cause several complications, such as urinary tract infections, bladder decompensation, and upper urinary tract deterioration with azotemia. The treatment modalities of BPH include medication and surgery. Currently, transurethral resection of the prostate (TUR-P) has been considered the gold standard of surgical treatment.^2^ The morbidity rate associated with TUR-P ranges from 15% to 20% and the mortality rate ranges from 0.2% to 2.5%.^3^ Recently, new surgical techniques and medical therapies, with benefits such as fewer bleedings, have been introduced and the number of laser prostatectomy have been increasing in these years. This procedure allows for potent delivery of heat to prostatic tissue through a laser fiber under cystoscopic vision. Only few articles have discussed the patient's preference and cost-effectiveness between laser prostatectomy and TUR-P. The high cost of equipment is one of the problems with laser therapy in the clinical practice. The cost-effectiveness of the outcome of using GreenLight HPS laser prostatectomy as a treatment for BPH was analyzed as well.

## MATERIALS AND METHODS

Between January 2005 and June 2011, all patients who came to Chang Gung Memorial Hospital to see K-HT, the corresponding author, with lower urinary tract symptoms and BPH were enrolled in this study. Their ages were between 50 and 93 (average 71.3) years. In total, the first 100 patients who were treated with TUR-P as well as the 100 patients treated with laser prostatectomy were reviewed. The single surgeon, K-HT, treated all 200 patients under spinal anesthesia. The inclusion criteria were: International Prostate Symptom Score of 15 or higher; a prostate sizing greater than 30 g on preoperative transrectal ultrasonography (TRUS) study; a peak flow rate of less than 12 mL/s; and a postvoiding residual volume of less than 500 mL. For the patients whose initial prostate-specific antigen (PSA) levels were above 4.0 ng/mL, a thorough TRUS evaluation and the prostate needle biopsy were performed in order to rule out the possibility of malignancy. Patients with evidence of prostate cancer or neurogenic bladder were excluded. The study (study code: 104–7627B) was approved by the Institutional Review Board at Chang Gung Memorial Medical center and written informed consent was obtained from each subject as a condition of entry.

The laser system used in this study was a high-powered 120W (GreenLight HPS) laser system and a side-firing AddStatTM fiber (American Medical System, Boston, MA) with a core diameter of 600 mm. Power settings were increased from 100 to 120 W after tissue was found to become resistant to vaporization.^4^

Uroflow and postvoiding residual volumes were evaluated before treatment and at 3, 6, 12, and 24 months after treatment. Before admission, the patients underwent TRUS of the prostate to determine any abnormalities. The TRUS of the prostate was performed with a Bruel and Kjaer ultrasonic scanner (model 1846, B & J Electronics, Copenhagen, Denmark) and a biplaner transrectal probe (model 8551). The prostate volume was calculated by multiplying the measurement of 3 dimensions at the largest cross-sectional area by a factor of 0.52. Upon admission to the hospital, the patient's complete history was recorded and a physical examination that included a digital rectal examination was performed. Results of laboratory examination of PSA level, urine analysis, complete blood count, chest x-ray, and electrocardiography were recorded. The results of treatment after laser prostatectomy were compared with the results of 100 patients who received TUR-P from the same surgeon.

### Calculating Costs

Total admission costs were divided into 6 categories: laboratory tests (eg, routine tests, serum chemistry, and PSA); radiological studies (eg, plain KUB x-ray, plain chest x-ray); pharmacological agents (all agents used during admission except those during anesthesia); operation and anesthesia (the operation fee and the agents used during anesthesia); others ward fees; and other charges related to treatment, (eg, bladder irrigation with normal saline, fleet enema, and perineal care).

### Outcome Analysis

Differences in admission charges of these 6 categories after HPS laser prostatectomy and TUR-P treatment were determined, and the results were compared between these 2 groups. The data were collected exclusively by the 1 author, analyzed by another and both were blind to each other.

### Statistical Analysis

The mean ± standard deviation of all continuous measures and scores were recorded at baseline and during all follow-up visits. Statistical analysis was performed using analysis of variance, or Student *t* test, to produce continuous data approximating normal distribution. Continuous variables were compared between treatment groups using a second Student *t* test with *P* values of 0.05 considered significant. Multivariate logistic regression analysis was performed to assess the impact of selected factors on postoperative outcome parameters, with *P* values of 0.05 considered statistically significant. All analyses were performed using commercially available software. The commercial statistical software SPSS V. 17 was used to analyze the data.

## RESULTS

### Patient Size and Characteristics

Table 1 displays the baseline characteristics of both patient groups. The laser prostatectomy group included patients of an older age (*P* = 0.25) with an American society of Anesthesiology score over 3 (*P* = 0.05), higher International Prostate Symptom Score (≧30) (*P* = 0.001), and high incidence of catheterization prior to the operation (*P* = 0.023). Additionally, the vast majority of patients who had thrombocytopenia underwent laser therapy (*P* < 0.05). The mean for operative duration of laser prostatectomy was higher than that of TUR-P, which showed that laser prostatectomy took longer than TUR-P (96.07 versus 68.4 minutes, *P* < 0.001). All objective urinary parameters showed significant improvement after laser prostatectomy. Table 2 displays the change in different urodynamic study parameters of the 100 patients at various stages after laser prostatectomy, which includes postvoid residual urine, peak flow rate, and average flow rate and voiding time, with standard deviation. The peak flow rate was increased by over 180% of the preoperative rate in the third month following HPS laser prostatectomy. The quality of life was initially 4.4 ± 1.1 and in 1 month was 2.15 ± 0.83, in 3 months was 1.94 ± 0.65, in 6 months was 1.77 ± 0.65, and 1.64 ± 0.92 in 12 months after surgery. These data showed a significantly improved quality of life in the patient group that chose laser prostatectomy.

**TABLE 1:**
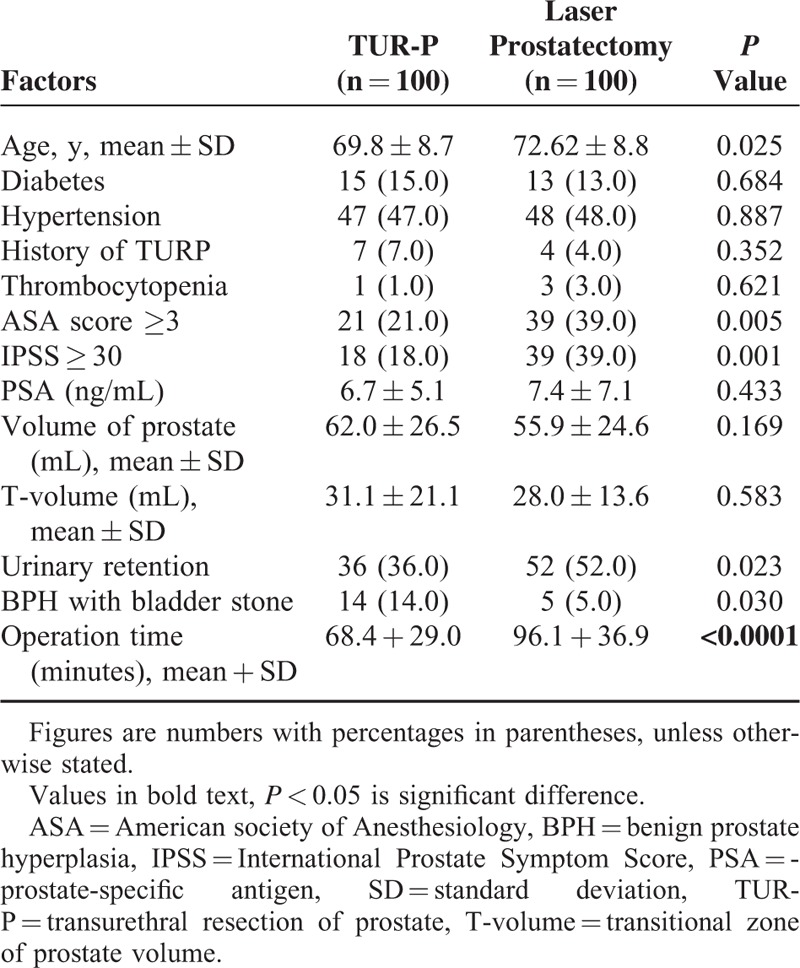
Baseline Characteristics

**TABLE 2:**
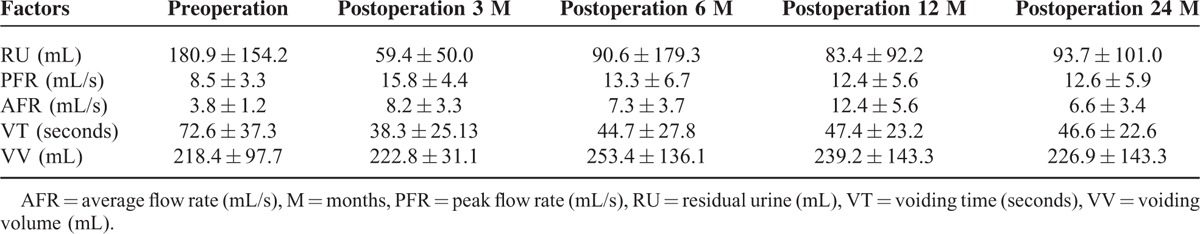
Urodynamic Results of Laser Prostatectomy in 100 Patients

Table 3 presents the intraoperative, early, and late postoperative complications and treated outcomes for patients in the both groups. No significant difference in the rate of complications was observed between the both groups. Nine patients using the urethral catheters for urinary retention presented with urinary retention after the laser surgery. Otherwise, the entire group of urinary retention patients was catheter free after 2 weeks. There was no significant difference between the laser and TUR-P group for the hematuria complication. The duration of stay in the hospital was significantly shortened in the laser group (5.3 ± 1.6 days versus 4.0 ± 2.1 days, *P* < 0.001).

**TABLE 3:**
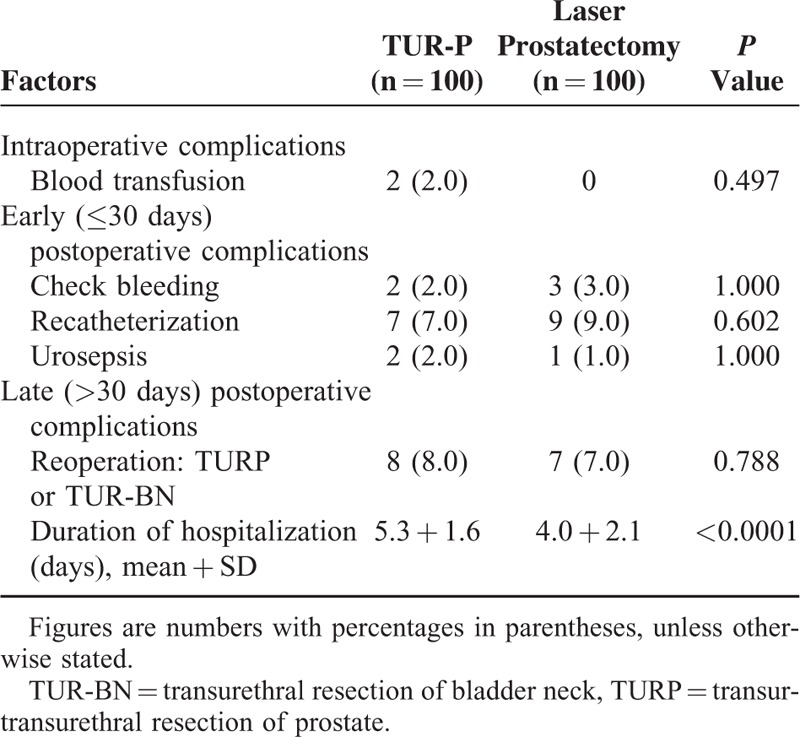
Intraoperative, Early, and Late Postoperative Complications and Outcomes in Patients in the Both Groups

In regard to the safety of laser surgery, we compared 2 groups: 1 group continued taking their antithrombotic medication, including antiplatelet such as aspirin and anticoagulants such as warfarin, for the laser surgery and another group ceased their antithrombotic medication before the TURP. Table 4 shows there were no significant differences in the perioperative complications, such as the need to check for bleeding, urinary retention rate, or urosepsis within 30 days after the surgery between both groups. The HPS laser group demonstrated high postoperative quality and safety even when the patient continued using antithrombotic medication during the laser surgery. The morbidity of these 2 using or not using antiplatelet (aspirin) medication demonstrated no significant difference between 2 groups postoperatively.

**TABLE 4:**
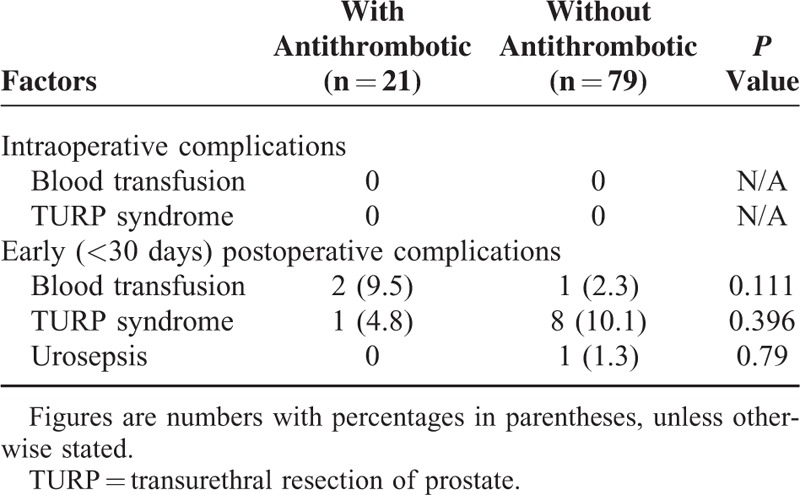
Intraoperative and Early Postoperative Complications and Outcomes in Patients Receiving Laser Prostatectomy Categorized According Using Antithrombotics or Not

### Outcome Analysis

Table 5 presents total cost to the hospital when comparing the laser and TUR-P groups. The mean stay in a hospital was significantly lower following laser treatment (*P* = 0.001). Although the total admission charges for the laser group exceeded those for the TUR-P group due to the cost of the laser equipment and accessories (laser fiber) (*P* < 0.001), the other admission charges were similar. Table 6 showed multivariate logistic regression analysis by choosing TUR-P as reference category. Operation time and duration of hospital stay are the only 2 independent variants during these 2 groups. Operation time is longer in laser prostatectomy group (odds ratio 1.026), whereas the duration of hospital stay is shorter in prostatectomy group (odds ratio = 0.493).

**TABLE 5:**
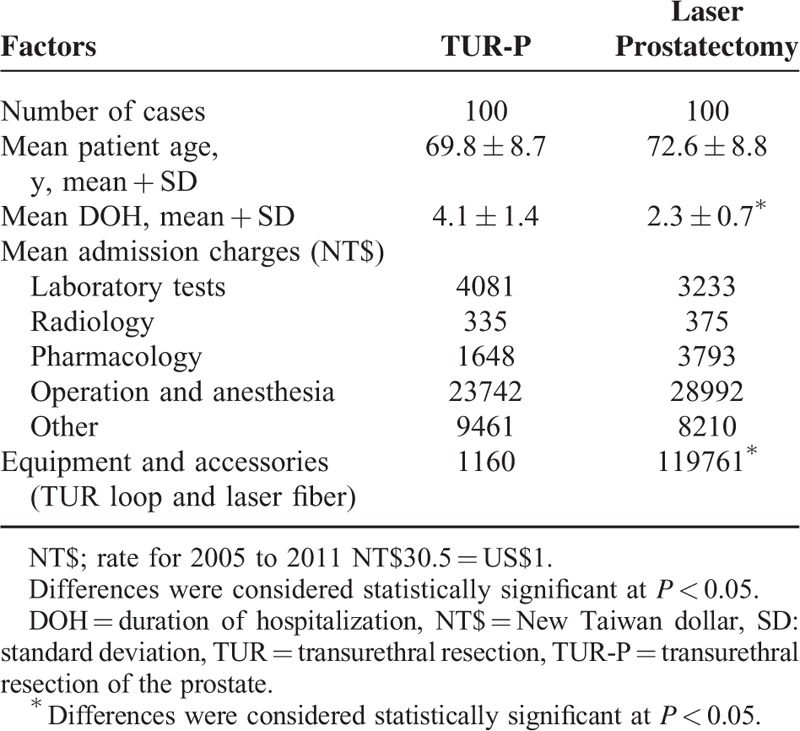
Details of Age, Duration of Hospitalization, and Admission Charges in Patients Categorized According to Experience of Attending Physician Between Implementation of the Transurethral Resection of Prostate and Laser Prostatectomy

**TABLE 6:**
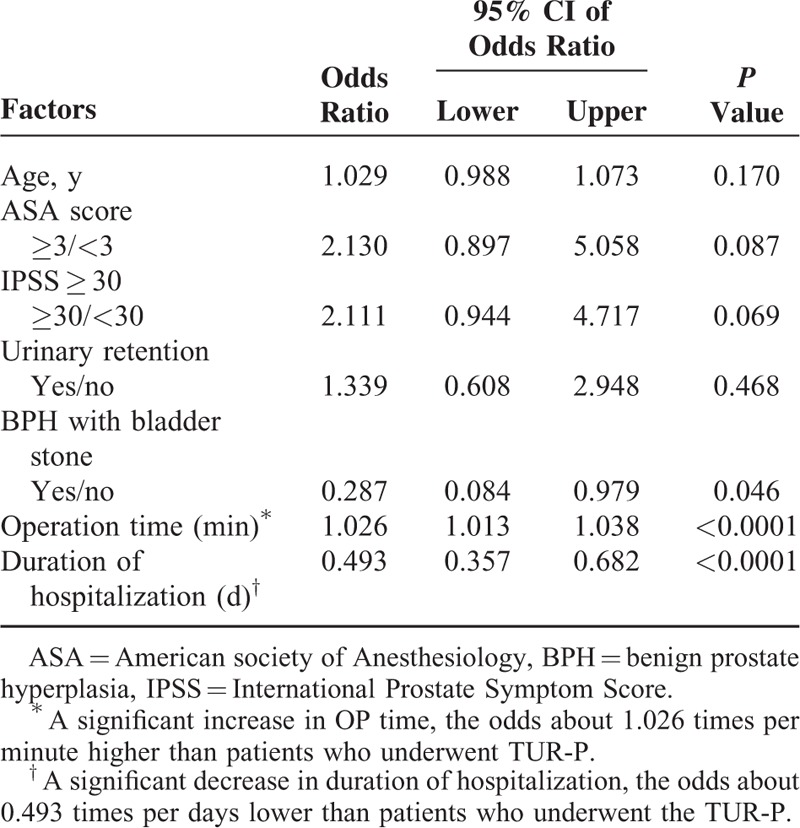
Multivariate Logistic Regression Analysis by Choosing Transurethral Resection of Prostate as Reference Category

## DISCUSSION

Laser prostatectomy was first introduced by Costello et al^5^ who used side-fire laser technology to establish a worthwhile outcome for BPH treatment.^6^ On treating Lower Urinary Tract Syndrome(LUTS), laser therapy provides outcomes and low complication rates at least noninferior to those obtained with TURP.^7,8^ In the last decade, laser prostatectomy has gradually become a popular treatment option for lower urinary tract symptoms caused by BPH. Side-fire laser devices vaporize the prostate urethra, along with the underlying prostate tissue. This results in immediate tissue removal with minimal bleeding and improved the peak flow rates.^7^ In this article, the therapeutic value and safety of laser prostatectomy were further examined, along with the cost-effectiveness of laser as an option for BPH treatment.

Of the procedural therapies studied, laser prostatectomy was more costly than TURP. The cost savings of this laser procedure stemmed from the rates of adverse events and retreatment, which on a comparative basis were lower for laser prostatectomy. Laser therapy can reduce the time of hospital stay when compared with the hospital stay after a TUR-P surgery. Thus, another benefit of laser therapy is that it increases the quality of life and creates minimal bleeding after surgery. Laser prostatectomy demonstrated the importance of cost-saving benefits of BPH procedural therapy for patients that result from fewer complications and postoperative bleeding.

In previous studies, the overall efficacy of laser therapy had been shown to be comparable with that of TUR-P.^9^ However, the disadvantage of laser prostatectomy includes: anesthesia is still required, voiding complaints accompanied with irritation may occur after treatment, and higher equipment cost.

The safety of the laser therapy had been reported in previous studies.^8^ It was observed that there was no significant difference in the perioperative bleeding rate in patients taking aspirin or warfarin compared with those who did not take these drugs. Due to the dangers of increased rates of cardiovascular and cerebrovascular complications when ceasing these medications, laser prostatectomy was found to be a safer alternative to TUR-P in treating patients taking antiplatelet and anticoagulation drugs.^8,10,11^ If the patient has a high surgical risk for bleeding and cardiovascular disease, laser therapy is a good alternative choice of treatment. The laser therapy yielded a higher preference among patients and improved the quality of life after surgery. When the aging males have to receive of TUR-P, 1 should always think about antiplatelet medication stopping, surgical mortality, and quality of life. The laser yielded a safer outcome than TUR-P due to the decrease in risk for the aging male. Otherwise, the disadvantage to this is the higher cost due to laser fiber equipment.^12^ When compared with patient surgical risk, patient preference and effect on the cardiovascular system, laser therapy showed significant improvement than the TUR-P procedure. Therefore, when the laser fiber cost, length of stay, comorbidity events, and medication costs are taken into account, the laser therapy is the better treatment than the TUR-P. Finally, the laser therapy will improve the quality of life, patient safety, and lower complications. The only disadvantage is the cost of the laser fiber.

## CONCLUSIONS

This study uses an innovative model to compare procedural treatment options for BPH. In comparison to TUR-P, laser prostatectomy is clinically effective but results in a high cost of treatment for symptomatic BPH due to the cost of the laser equipment. Laser prostatectomy is a more sophisticated and acceptable modality for the treatment of BPH due to the efficiency, short learning curve, and low morbidity profile of using laser prostatectomy. However, since the laser is a new technology, the expense, safety precautions, long-term effectiveness, and general acceptance are all important limiting factors. Future studies should include a larger sample and prolonged follow-up of patients undergoing laser prostatectomy.
